# A Porcine Circovirus Type 2 (PCV2) Mutant with 234 Amino Acids in Capsid Protein Showed More Virulence *In Vivo*, Compared with Classical PCV2a/b Strain

**DOI:** 10.1371/journal.pone.0041463

**Published:** 2012-07-19

**Authors:** Longjun Guo, Yujie Fu, Yiping Wang, Yuehua Lu, Yanwu Wei, Qinghai Tang, Peihu Fan, Jianbo Liu, Long Zhang, Feiyan Zhang, Liping Huang, Dan Liu, Shengbin Li, Hongli Wu, Changming Liu

**Affiliations:** Division of Swine Infectious Diseases, State Key Laboratory of Veterinary Biotechnology, Harbin Veterinary Research Institute, Chinese Academy of Agricultural Sciences, Harbin, China; Institut Pasteur, France

## Abstract

**Background:**

Porcine circovirus type 2 (PCV2) is considered to be the primary causative agent of postweaning multisystemic wasting syndrome (PMWS), which has become a serious economic problem for the swine industry worldwide. The major genotypes, PCV2a and PCV2b, are highly prevalent in the pig population and are present worldwide. However, another newly emerging PCV2b genotype mutant, which has a mutation in its ORF2-encoded capsid protein, has been sporadically present in China, as well as in other countries. It is therefore important to determine the relative virulence of the newly emerging PCV2b genotype mutant, compared with the existing PCV2a and PCV2b genotypes, and to investigate whether the newly emerging mutant virus induces more severe illness.

**Methodology/Principal Findings:**

Twenty healthy, 30-day-old, commercial piglets served as controls or were challenged with PCV2a, PCV2b and the newly emerging mutant virus. A series of indexes representing different parameters were adopted to evaluate virulence, including clinical signs, serological detection, viral load and distribution, changes in immune cell subsets in the peripheral blood, and evaluation of pathological lesions. The newly emerging PCV2 mutant demonstrated more severe signs compatible with PMWS, characterized by wasting, coughing, dyspnea, diarrhea, rough hair-coat and depression. Moreover, the pathological lesions and viremia, as well as the viral loads in lymph nodes, tonsils and spleen, were significantly more severe (P<0.05) for piglets challenged with the newly emerging mutant compared with those in the groups challenged with PCV2a and PCV2b. In addition, a significantly lower average daily weight gain (P<0.05) was recorded in the group challenged with the newly emerging PCV2 mutant than in the groups challenged with the prevailing PCV2a and PCV2b.

**Conclusions:**

This is believed to be the first report to confirm the enhanced virulence of the newly emerging PCV2 mutant *in vivo*.

## Introduction

Porcine circovirus type 2 (PCV2) is considered generally to be the primary causative agent of postweaning multisystemic wasting syndrome (PMWS), which has become a serious economic problem for the swine industry worldwide. The various clinical manifestations of PCV2 infection in pigs across the age groups have become known as PCVAD, and are characterized by wasting and growth retardation [1]. PCV2 is a small, single-stranded, ambisense DNA virus [2]–[3] that belongs to the genus *Circovirus* in the family *Circoviridae* [4]. PCV2 contains two major open reading frames (ORFs), oriented in opposite directions, which encode proteins associated with replication (ORF1, 945 nt) and the virus capsid (ORF2, 702 nt) [5]–[6]. Recent phylogenic investigations have indicated that PCV2 can be subdivided further into several types, of which PCV2a and PCV2b are highly prevalent in the pig population and are present worldwide [7]–[9]. PCV2 has undergone much genetic variation in recent years [10]–[19]. In addition, a newly emerging PCV2 mutant with an additional lysine (K) in the ORF2-encoded capsid protein has been isolated from a sample from an aborted pig with PMWS in China, and identification has been performed *in vitro* [17]. In the newly emerging PCV2 mutant, a shift from TTA to CTT in the genomic sequence resulted in a mutation of the stop codon (from UAA to AAG) in ORF2, to give an ORF2 gene of 705 nt with another stop codon [20]. However, information regarding differences in pathogenicity between the newly emerging PCV2 mutant and the main prevailing genotypes PCV2a and PCV2b, as well as the correlation between the mutation and pathogenicity, remains limited.

The objective of this research was to assess the relative virulence of the newly emerging PCV2 mutant PCV2b/rBDH, recovered in 2008 from a sample from an aborted pig with PMWS, when compared with PCV2a/rCL and PCV2b/rJF, representative strains of different PCV2 genotypes, respectively.

## Results

### Clinical examination

**Figure 1:**
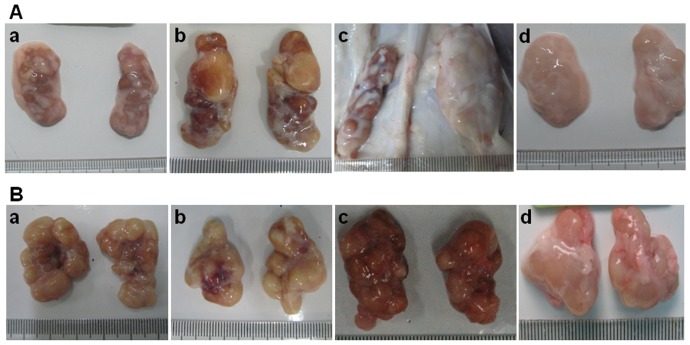
Clinical pictures of inguinal (A) and submandibular (B) lymph nodes in the different challenge groups. Moderate to severe atrophy, enlargement and hemorrhage were observed in the inguinal and submandibular lymph nodes, respectively, compared with the control group. More severe clinical signs were observed in the PCV2b/rBDH-challenged group, such as simultaneous presence of atrophy, enlargement and hemorrhage of inguinal and submandibular lymph nodes. a: PCV2a/rCL challenge group; b: PCV2b/rJF challenge group; c: PCV2b/rBDH challenge group; d: control group.

No apparent gross lesions were observed in the control group; however, clinical signs compatible with PMWS, characterized by wasting, coughing, dyspnea, diarrhea, rough hair-coat and depression, were observed mainly from 10 to 31 days post-challenge (DPC). The mean clinical score (CS) was significantly higher (P<0.05) in the PCV2b/rBDH challenged group compared with those of the PCV2a/rCL challenged group and the PCV2b/rJF challenged group throughout the study period, which confirmed the greater virulence of the newly emerging mutant PCV2b/rBDH in piglets than that associated with the classical PCV2a and PCV2b genotypes. Based on the CS in the piglets, however, no significant difference was demonstrated between the PCV2a/rCL and PCV2b/rJF challenged groups (P>0.05). The detailed CS values are shown in Table 1. With regard to the rectal temperature, in both the challenged groups and in the control group, the rectal temperatures of the piglets did not exceed 40°C up to the termination of the experiment. However, significantly more severe clinical manifestations were demonstrated in the PCV2b/rBDH challenged group compared with those induced in the remaining challenged groups and the control group. For example, there were more severe pathological lesions in the inguinal and submandibular lymph nodes (Figure 1).

**Table 1:** Scored values for clinical condition and pathological lesions for piglets in different challenge groups and the control group.

	PCV2a/rCL	PCV2b/rJF	PCV2b/rBDH	Control
**Physical condition**				
Wasting	1, 1, 1, 1, 1	1, 1, 1, 1, 1	2, 2, 2, 1, 2	0, 0, 0, 0, 0
Coughing	1, 0, 0, 1, 1	1, 1, 0, 1, 1	1, 2, 2, 2, 2	0, 0, 0, 0, 0
Rough hair-coat	1, 1, 1, 1, 1	1, 1, 1, 2, 1	2, 2, 2, 2, 2	0, 0, 0, 0, 0
Dyspnea	0, 0, 1, 1, 0	1, 0, 1, 1, 2	1, 2, 2, 1, 1	0, 0, 0, 0, 0
Diarrhea	0, 1, 1, 0, 0	0, 0, 0, 0, 1	0, 1, 2, 1, 1	0, 0, 0, 0, 0
Depression	1, 1, 1, 1, 1	1, 1, 1, 1, 1	2, 1, 1, 2, 2	0, 0, 0, 0, 0
Median score	4	5	10	0
**Pathological condition**				
Ascites or edema	0, 0, 0, 0, 0	0, 0, 0, 0, 0	0, 0, 0, 2, 0	0, 0, 0, 0, 0
Icterus	0, 0, 0, 0, 0	0, 0, 1, 0, 0	0, 2, 0, 0, 1	0, 0, 0, 0, 0
Liver pale or congested	0, 1, 0, 0, 0	1, 0, 0, 1, 0	1, 2, 0, 0, 1	0, 0, 0, 0, 0
Atrophy of lymph nodes	1, 0, 1, 1, 0	1, 1, 1, 0, 1	2, 2, 2, 1, 2	0, 0, 0, 0, 0
Enlargement and hemorrhage of peripheral lymph nodes	1, 0, 0, 1, 1	1, 0, 2, 1, 0	2, 2, 1, 1, 1	0, 0, 0, 0, 0
Median score	1	2	5	0
**Histological lesions**				
***Lymphocyte depletion***				
Inguinal lymph node	1, 2, 1, 2, 1	2, 1, 1, 3, 1	2, 3, 3, 3, 3	0, 0, 0, 0, 0
Submandibular lymph node	1, 1, 1, 1, 1	1, 1, 2, 1, 1	3, 3, 1, 2, 3	0, 0, 0, 0, 0
Mesenteric lymph node	1, 1, 1, 1, 1	1, 2, 1, 1, 2	2, 2, 3, 3, 3	0, 0, 0, 0, 0
Tonsil	1, 1, 1, 1, 1	2, 1, 2, 3, 1	1, 3, 2, 3, 2	0, 0, 0, 0, 0
***Granulomatous inflammation***				
Inguinal lymph node	1, 1, 1, 1, 1	1, 1, 1, 1, 2	1, 3, 3, 2, 3	0, 0, 0, 0, 0
Submandibular lymph node	1, 2, 1, 1, 1	1, 1, 1, 1, 1	3, 3, 1, 2, 3	0, 0, 0, 0, 0
Mesenteric lymph node	1, 1, 1, 1, 0	1, 0, 1, 1, 3	1, 3, 3, 2, 1	0, 0, 0, 0, 0
Tonsil	1, 1, 0, 1, 1	2, 1, 1, 0, 1	1, 1, 2, 3, 2	0, 0, 0, 0, 0
Lung (inflammatory infiltration)	1, 0, 0, 1, 1	1, 1, 1, 1, 1	1, 2, 2, 1, 3	0, 0, 0, 0, 0
Median score	9	12	21	0
**Total median score**	15	20	34	0

### Average daily weight gain (ADWG)

**Figure 2:**
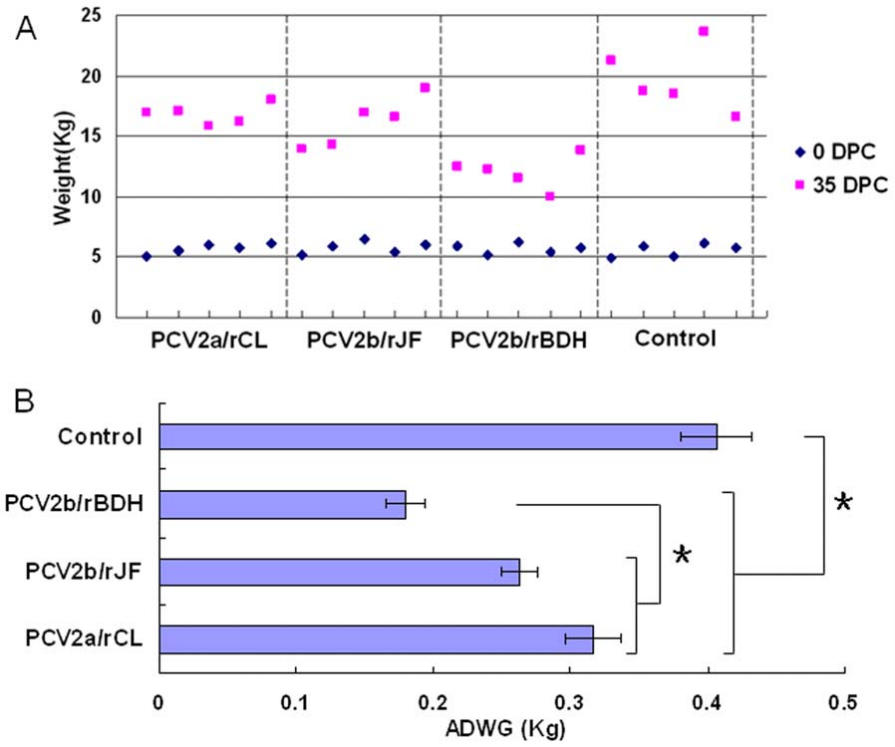
Weight of individual animals from each group (A) and ADWG (B). A: Weight of individual animals from each group at days 0 and 35, respectively. B: ADWG±SD was calculated for each treatment group throughout the experiment, with (*) indicating significant differences between the specified pairs. ADWG was significantly higher in the control group compared with the challenged groups (P<0.05). A significantly lower ADWG was observed in the PCV2b/rBDH-challenged group than in both the PCV2a/rCL- and PCV2b/rJF-challenged groups (P<0.05). No significant difference was seen between the PCV2a/rCL- and PCV2b/rJF-challenged groups (P>0.05).

As shown in Figure 2, the ADWG was higher in the control group than in the challenge groups, and this difference was found to be statistically significant (P<0.05). Moreover, a significantly lower ADWG was observed in the PCV2b/rBDH-challenged group than in the other two challenge groups (P<0.05), which could represent persuasive proof of the greater virulence of the PCV2b/rBDH mutant. Meanwhile, no significant difference was found between the PCV2a/rCL- and PCV2b/rJF-challenged groups on the basis of the ADWG (P>0.05).

### Viremia

For the detection of viremia, viral DNA was extracted from serum samples collected at 0, 3, 7, 10, 14, 21, 28 and 35 DPC and investigated using routine PCR. No viremia was detected in the piglets of the control group; in contrast, all piglets with PCV2 infection showed viremia, which persisted for about 7 days from around 21 to 28 DPC in the challenge groups. Earlier and more severe viremia was observed in the PCV2b/rBDH-challenged group compared with the other two challenge groups. However, no viremia was observed after 35 DPC (Table 2).

**Table 2:** Detection of PCV2 viral DNA using PCR in sera of pigs challenged experimentally with different PCV2 strains at different time points post challenge.

Group	Days post challenge							
	0	3	7	10	14	21	28	35
PCV2a/rCL	0/5^a^	0/5	0/5	0/5	0/5	4/5	5/5	0/5
PCV2b/rJF	0/5	0/5	0/5	0/5	0/5	4/5	5/5	0/5
PCV2b/rBDH	0/5	0/5	0/5	0/5	2/5	5/5	5/5	0/5
Control	0/5	0/5	0/5	0/5	0/5	0/5	0/5	0/5

a : Number of PCV2 positive/Number of pigs tested.

### Detection of PCV2-specific antibody

The immunoperoxidase monolayer assay (IPMA) was used to investigate the level of PCV2-specific antibodies in the sera of piglets from the PCV2-challenged groups and the control group. As shown in Table 3, PCV2-specific antibodies appeared around 4 weeks post-challenge in all the challenge groups, and the antibody titers increased slightly, except in the PCV2a/rCL-challenged group, which showed a slight decrease. In contrast, no PCV2-specific antibodies were detected in the control group.

**Table 3:** Titers of PCV2-specific antibodies in sera at different time points.

Group Name	Antibody titration on different days post challenge (DPC)							
	0	3	7	10	14	21	28	35
PCV2a/rCL	<25	<25	<25	<25	<25	<25	100	50∼100^a^
PCV2b/rJF	<25	<25	<25	<25	<25	<25	50	50∼100^b^
PCV2b/rBDH	<25	<25	<25	<25	<25	<25	100	100∼200^c^
Control	<25	<25	<25	<25	<25	<25	<25	<25

a: one piglet was 50 and the other four piglets were all 100.

b: two piglets were 50 and the other three piglets were all 100.

c: one piglet was 100 and the other four piglets were all 100.

### Virus distribution and quantification by real-time quantitative PCR (qPCR) in different organs/tissues

**Figure 3:**
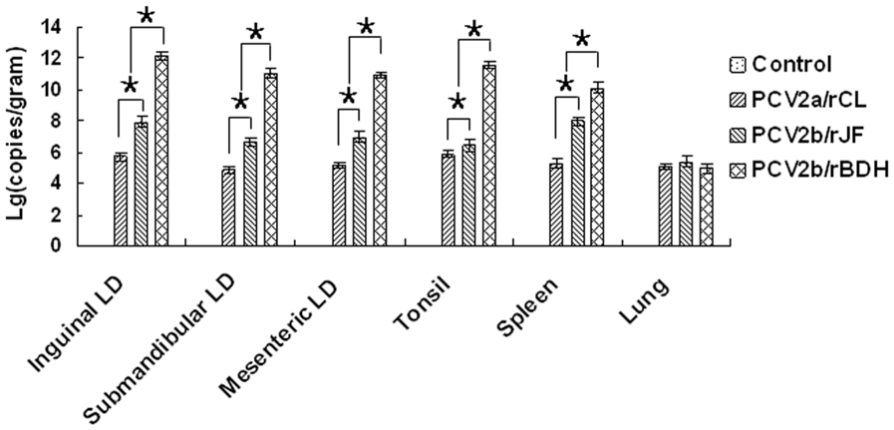
Quantification and distribution of viral DNA loads. Detection and quantification of viral DNA loads in lymphoid tissues, as well as spleen and lung, in pigs challenged experimentally with the PCV2a/rCL, PCV2b/rJF or PCV2b/rBDH virus. Group mean logarithm viral genomic copies/gram of tissue (±SD) was calculated as the corresponding value on the *x* axis for each treatment group. All samples from MEM-challenged pigs were negative. Pairs of treatments with (*) were significantly different (P<0.05).

PCV2 was localized mainly in the inguinal, submandibular and mesenteric lymph nodes, tonsils, spleen and lungs. The viral loads in all the organs/tissues investigated (except lung) of the PCV2b/rBDH-challenged group were significantly higher than those in the remaining challenge groups and the control group (P<0.05). In addition, a significantly greater viral load was present in all organs/tissues investigated in the PCV2b/rJF- than in the PCV2a/rCL-challenged group (P<0.05), except for the lungs (P>0.05). There was no difference in the viral loads present in the lungs of the three challenge groups (P>0.05). The results of qPCR performed on the organ/tissue samples are summarized in Figure 3.

### Pathological lesions

**Figure 4:**
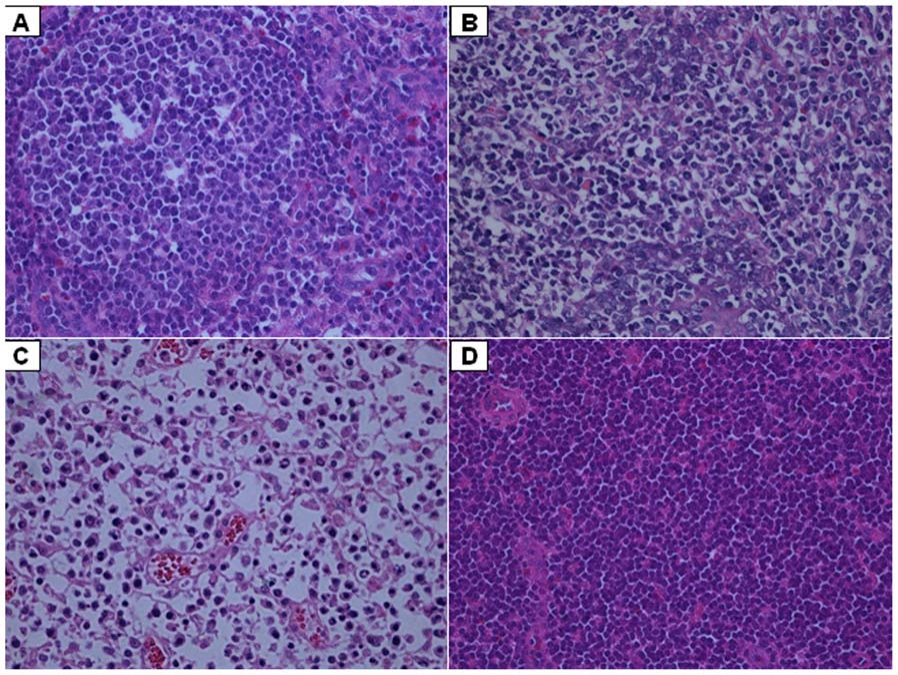
Histopathological detail of inguinal lymph nodes showing different degrees of severity of lymphocyte depletion. (A) Inguinal lymph node from PCV2a/rCL-challenged group with mild lymphocyte depletion. (B) Inguinal lymph node from PCV2b/rJF-challenged group with moderate lymphocyte depletion. (C) Inguinal lymph node from PCV2b/rBDH-challenged group with significant lymphocyte depletion. (D) Normal inguinal lymph node from control group with normal lymphocyte count. Hematoxylin & eosin staining (400×).

The macropathological lesions (i.e. the pathological condition) and the micropathological lesions (i.e. histological lesions) are referred to together as the pathological lesions. The score for the pathological condition was significantly higher in the PCV2b/rBDH-challenged group than in the other two challenge groups (P<0.05), which indicated that PCV2b/rBDH showed greater virulence. The histological lesions in the inguinal, submandibular and mesenteric lymph nodes, tonsils, spleens and lungs were examined. No apparent gross lesions were observed in the control group. In contrast, in the PCV2-challenged groups, the piglets showed moderate to severe gross lesions in the inguinal, submandibular and mesenteric lymph nodes, tonsil, spleen and lungs, which were consistent with a diagnosis of PMWS. When evaluated using the scores from the pathological condition or the histological lesions, more severe lesions were induced in the PCV2b/rBDH-challenged group than in the other two challenge groups. Additionally, the score was significantly higher in the PCV2b/rJF-challenged group than that in the PCV2a/rCL-challenged group on the basis of the histological lesions (P<0.05), whereas no significant difference was demonstrated between the PCV2a/rCL- and PCV2b/rJF-challenged groups on the basis of the score for the pathological condition (P>0.05). All three scores were higher (P<0.05) in the PCV2-challenged groups than in the control group. We speculate that the newly emerging mutant PCV2b/rBDH showed greater virulence than the classical PCV2a and 2b genotypes, on the basis of the significantly higher scores (P<0.05). The results are summarized in Table 1. In addition, more severe lesions were observed in almost all the organs/tissues investigated in the PCV2b/rBDH-challenged group than in the other groups, on the basis of micropathological examination, for example, more severe depletion of lymphocytes in the inguinal lymph nodes (Figure 4).

### Immune cell subsets in the peripheral blood

**Figure 5:**
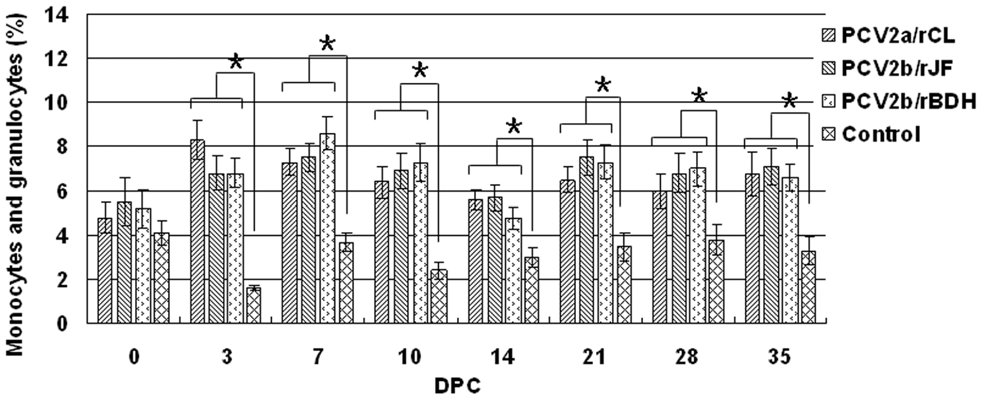
Proportion of monocytes and granulocytes in leukocyte subpopulations. The proportion of monocytes and granulocytes in leukocyte subpopulations was significantly higher in the three PCV2 challenge groups compared with that in the control group, but no significant differences were observed among the three PCV2 challenge groups. Pairs of treatments with (*) were significantly different (P<0.05).

**Figure 6:**
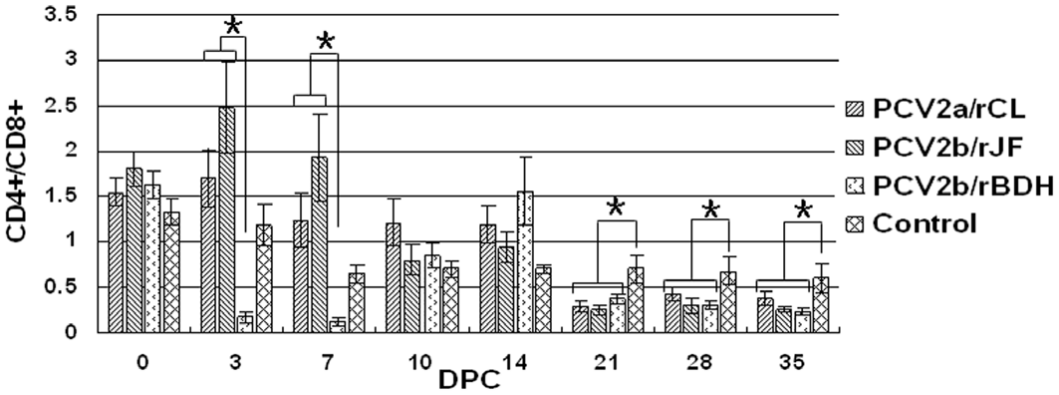
Changes of CD4^+^/CD8^+^ cells in peripheral blood of infected piglets. Ratio of CD3^+^CD4^+^CD8^−^ cells to CD3^+^CD4^−^CD8^+^ cells (CD4^+^/CD8^+^) in PCV2b/rBDH-challenged group was significantly lower than that of the other two challenge groups at 3 and 7 DPC (P<0.05). When evaluated as a whole, all the PCV2 challenge groups showed a significant continuous decrease in the CD4^+^/CD8^+^ ratio, when compared with the control group from 21 DPC. Pairs of treatments with (*) were significantly different (P<0.05).

The proportions of monocytes and granulocytes in the leukocyte subpopulations was significantly higher in the three PCV2 challenge groups compared with that in the control group (P<0.05), but no significant difference was observed among the three PCV2 challenge groups (Figure 5). The ratio of CD3^+^CD4^+^CD8^−^ cells to CD3^+^CD4^−^CD8^+^ cells (CD4^+^/CD8^+^) in the PCV2b/rBDH-challenged group was significantly lower than that of the other two challenge groups at 3 and 7 DPC, respectively (P<0.05). In addition, when evaluated as a whole, all the PCV2 challenge groups showed a significant continuous decrease in the CD4^+^/CD8^+^ ratio when compared with the control group, from 21 DPC, which indicated an increase in the number of CD8^+^ cells relative to CD4^+^ cells (Figure 6).

### Sequence confirmation

The DNA sequences amplified from the inguinal lymph nodes were the same as those used for challenge in different groups. It was confirmed that no other PCV2 virus contamination was present in this study (data not shown).

## Discussion

PCV2 is the major swine pathogen associated with PCV-associated diseases, including PMWS [21]. The molecular mechanisms of PCV2 replication and pathogenesis are poorly understood, but PMWS associated with PCV2 infection has become an economically important global disease [22]. This is believed to be the first report of a newly emerging PCV2 mutant with significantly enhanced virulence, when compared with the main prevailing genotypes PCV2a and PCV2b.

The newly emerging PCV2 mutant was isolated in 2008 from a serum sample from an aborted pig with PMWS. In addition, Knell et al. [23] have reported previously that mutations could occur in the ORF2 gene, because a deletion (T) was found at position 1042 in the 1767 nt genome of one strain (GenBank no. AY713470), which led to elongation by one amino acid (lysine) in the C terminus of the ORF2-encoded Cap protein. Olvera et al. [15] have also reported elongation by one lysine (K) residue of the C terminus of the Cap protein due to a mutation in the stop codon of ORF2. However, information remains limited regarding differences in pathogenicity between the newly emerging PCV2 mutants with capsid protein mutations and the two current main prevailing genotypes PCV2a and PCV2b, as well as the correlation between mutation and pathogenicity. In this study, we confirmed first, by experiments with pigs, that the newly emerging PCV2 mutant obtained in our laboratory showed significantly enhanced virulence *in vivo*. Overall, we found a more severe pathogenicity caused by the newly emerging PCV2b mutant than the classical PCV2a and PCV2b genotypes, and no significant difference in pathogenicity was observed between the classical PCV2a and PCV2b; these results confirm differences in virulence among PCV2 strains. To date, experimental infection models analyzing differences in virulence have reported mixed results [24]. In one previous study involving inoculation of gnotobiotic pigs with infectious DNA clones derived from PCV2a or PCV2b, differences in symptom onset and overall mortality were reported [25]. Other results suggest that PCV2 isolates differ in virulence in a specific pathogen free (SPF) pig model. There are marked differences in the clinical expression of diseases associated with PCV2 in the field [26]. Moreover, results from the same laboratory have indicated that the virulence of PCV2a and PCV2b isolates does not differ in the conventional SPF pig model; however, the virulence of isolates within the same cluster does differ. The increased virulence that is associated with PCV2b isolates in the field was not observed under the conditions of the present study [27]. In addition, Harding et al. have indicated that dual heterologous PCV2a/2b inoculation may induce severe clinical illness, but PCV2a and PCV2b when administered singularly appear to be of equivalent virulence [28]. Taking the previous results together, different PCV2 genotypes or clusters may demonstrate diversity in clinical pathogenicity and virulence, and other PCV2 with more virulence would be present under the natural selection pressure as the PCV2 evolution and variation. Based on the putative ORF2-encoded amino acid analysis, the ORF2 of the newly emerging PCV2 mutant encodes a capsid protein with 234 aa, which is one lysine residue (K) more than that of the classical PCV2a and PCV2b genotypes, which have 233 aa. The increased virulence of the newly emerging PCV2b/rBDH could have resulted from the addition of the lysine (K) to the ORF2-encoded Cap protein. In the future, other PCV2 mutants may emerge that result from genetic evolution and selection of PCV2 under environmental pressures, and these may exhibit enhanced virulence.

The significance of PCV2 mutations in terms of PCV-associated pathogenesis is unclear. The virulence properties of PCV2 appear to be located in the capsid protein. For example, it has been reported that the attenuation that occurred following serial passage of an isolate of PCV2 was the result of mutations in the capsid protein [22]. However, the same group reported that a chimera incorporating ORF1 from PCV1 and ORF2 from PCV2 was non-pathogenic in pigs [29]. It is not a single ORF1 gene or ORF2 gene that contributes to changes in the virulence and pathogenicity of PCV2, but the involvement of the whole virus genome [30]. Therefore, mutation of the capsid protein may contribute to enhanced virulence, but may not be the only factor. The findings of this study may facilitate future studies on the differences in pathogenicity between the existing major genotypes (PCV2a and PCV2b) and new PCV2 mutants, and may contribute to a deeper understanding of the molecular epidemiology of PCV2. The significance of the newly emerging PCV2 mutant requires further investigation, and additional studies are underway to determine how the newly emerging PCV2 mutant acts in enhancing the virulence of PCV2.

## Materials and Methods

### Cells and viruses

**Figure 7:**
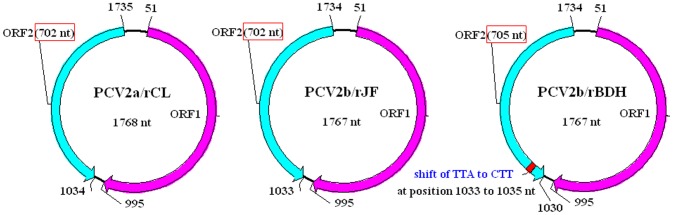
Schematic diagram of PCV2 strains used in this study. For the PCV2b/rBDH strain, a shift from TTA to CTT in the genomic sequence resulted in a stop codon mutation (from UAA to AAG) in ORF2 (in the antisense chain of the genomic sequence of PCV2), giving an ORF2 gene of 705 nt with another stop codon, compared with the classical PCV2a and PCV2b genotypes.

The porcine kidney cell line (PK15), free of PCV1 contamination, was obtained by successive subcloning from the American Type Culture Collection (ATCC CCL-33). It was maintained at 37°C with 5% CO_2_ in minimum essential medium (MEM) (Invitrogen, Carlsbad, CA, USA) supplemented with 5% heat-inactivated fetal bovine serum (FBS), 100 IU/mL penicillin and 100 µg/mL streptomycin. Different genotypes of representative strains of PCV2, which were termed PCV2a/CL, PCV2b/JF and PCV2b/BDH, with GenBank accession numbers JF682791, JF682792 and HM038017, respectively, were isolated and maintained in the Harbin Veterinary Research Institute, Chinese Academy of Agricultural Sciences. Three corresponding cloned strains with a molecular marker, termed PCV2a/rCL, PCV2b/rJF and PCV2b/rBDH, were obtained and used as animal-challenge strains in this study to avoid contamination of the parental viruses with other unknown viruses [20]. The ORF2 of the newly emerging PCV2 mutant encodes its capsid protein, which has 234 aa, one amino acid more than that of the classical PCV2a and PCV2b genotypes with 233 aa (Figure 7). Detailed information on the cloned PCV2 viruses is summarized in Table 4. Experimental infection of animals was performed with the 10^th^ passage of these three viruses in culture, with the virus infectious dose adjusted to 10^4.5^ TCID_50_/mL.

**Table 4:** Characteristics of PCV2 strains used for the infection experiment in this study.

Characteristics	PCV2 strains		
	PCV2a/rCL	PCV2b/rJF	PCV2b/rBDH
GenBank No	HM038033	HM038022	HM038017
Genotype	PCV2a	PCV2b	PCV2b
PassageΔ	10	10	10
Year of isolation	2008	2008	2008
Genome length (nt)	1768	1767	1767
ORF2 (aa)	233	233	234
ORF1 (aa)	314	314	314
Number of amino acids*	0	0	1 (K)

Note:

Δ represents the number of passages of the PCV2 strains used for the infection experiment in this study;

* represents the number of additional amino acids encoded by ORF2 compared with classical PCV2 strains with 233 aa in the ORF2-encoded Cap protein.

### Animals

Twenty healthy, weaned, 30-day-old, commercial Large White–Dutch Landrace crossbred piglets were obtained from a pig farm that was negative for PCV2 and porcine reproductive and respiratory syndrome virus (PRRSV) infections using commercial enzyme-linked immunosorbent assay (ELISA) kits for PRRSV antibody detection (IDEXX Labs Inc., Westbrook, ME, USA) and PCV2 antibody detection (INGEZIM CIRCOVIRUS IgG/IgM kit; Ingenasa, Madrid, Spain) and using RT-PCR or PCR for viral nucleic acid detection. Using serological methods and/or RT-PCR/PCR, the animals were shown to be negative for classical swine fever virus, porcine parvovirus, pseudorabies virus ,swine influenza virus and *Mycoplasma hyopneumoniae* infections prior to this study. The animal experiment was approved by Harbin Veterinary Research Institute and performed in accordance with animal ethics guidelines and approved protocols. The animal Ethics Committee approval number is Heilongjiang-SYXK-2006-032.

### Experimental design

The 30-day-old piglets were transported to the national level 2 animal facilities at the Harbin Veterinary Research Institute. The pigs were divided randomly into three challenge groups and a control group (five piglets per group) and were raised separately in different isolation rooms with individual ventilation. The animals received food and water *ad libitum*. Each pig in each challenge group was inoculated both intranasally and intramuscularly with 1 mL viral culture using the cloned PCV2 strains PCV2a/rCL, PCV2b/rJF and PCV2b/rBDH, respectively; each milliliter contained 10^4.5^ TCID_50_ from the 10^th^ passage on PK15 cells. Each pig in the control group was inoculated with the same volume of MEM, using the same inoculation route and procedure mentioned above. On the day of challenge and at the end of the experiment, all the piglets were weighed, and the ADWG (kg/day), expressed as (body weight at 35 DPC – body weight at 0 DPC)/35, was determined. Blood samples were collected for virological and serological examination at 0 (before inoculation), 3, 7, 10, 14, 21, 28 and 35 DPC. EDTA-stabilized blood samples were collected simultaneously for flow cytometry (FCM) analysis for T cells, and monocyte and granulocyte subpopulations, at 0, 7, 10, 14, 21, 28 and 35 DPC. After challenge, all piglets were examined clinically and their rectal temperatures were taken daily during the experimental period. At 35 DPC, the trial was terminated and all piglets were killed, postmortem examinations were performed, and gross lesions were recorded, followed by pathological examination.

### Clinical examination

Piglets of the abovementioned PCV2-challenged groups and the control group were examined clinically and a physical condition score was assessed weekly. The clinical signs evaluated included the presence of respiratory and digestive clinical signs (wasting, coughing, dyspnea and diarrhea), behavior (depression) and pathological condition. The clinical parameters were scored using a numeric value ranging between 0 and 2 (none, 0; mild, 1; severe, 2) [31]. The clinical examination of physical condition was recorded daily in detail from 10 to 31 DPC and the final score was given after overall evaluation during this period by a blinded researcher. On the basis of the recorded parameters, a CS was calculated using the formula: CS = [(numeric value of wasting + numeric value of coughing + rough hair-coat + numeric value of dyspnea + numeric value of diarrhea + numeric value of behavior)].

### Serology to detect PCV2 antibodies

Serum samples obtained throughout the study at 0, 3, 7, 10, 14, 21, 28 and 35 DPC were used to determine the presence of specific antibodies against PCV2, using an IPMA, as described by Liu et al. [32]. The IPMA was used to detect changes with time in PCV2-specific antibody *in vivo* post PCV2 challenge. Briefly, 96-well plates containing PCV2a/LG and mock-infected cells were fixed in 33% acetone–PBS for 20 min at room temperature and dried for testing serum samples. Each serum sample was first diluted 25-fold and then twofold serially diluted in PBS. The diluted serum samples, PCV2-positive sera and PCV2-negative sera were added to PCV2/LG, and mock-infected cells, respectively, and then incubated at 37°C for 1 h. After the unbound antibodies were washed three times with PBS, a 1∶3,000 dilution of HRP-conjugated Protein A (Invitrogen) as a secondary antibody was added and incubated for 1 h at 37°C. After washing, color development was carried out with 3-amino-9-ethylcarbazole and hydrogen peroxide in 0.05 M acetate buffer (pH 5.0) for 30 min at 37°C. The reaction was terminated by removal of the substrate. Plates were examined under an inverted light microscope. The antibody titers were calculated as the reciprocal of the last dilution at which positive cells were detected.

### Detection of PCV2 viremia in serum samples by PCR

Serum samples were collected from all animals at 0, 3, 7, 10, 14, 21, 28 and 35 DPC, respectively, and PCV2 nucleic acid was detected with a pair of PCR procedures described previously [33]. Briefly, a pair of primers, D1 (959–979 nt; 5′- CCCATGCCCTGAATTTCCATA -3′) and D2 (1311–1290 nt; 5′- TAAACTACTCCTCCCGCCATAC -3′) was used for amplification to give a product 353 bp in length, and viral DNA was extracted from the serum samples using a DNA extraction kit (Tiangen, Beijing) according to the manufacturer's instructions. The amplification was performed in a 25-µL reaction mixture containing 5 µL KOD DNA polymerase reaction buffer (TOYOBO Biotechnology Co. Ltd., Shanghai), 0.1 mM each dNTP, 0.4 µM each primer, 1 µL (1 U) KOD-Plus-Ver.2 high fidelity DNA polymerase (TOYOBO Biotechnology Co. Ltd.) and 2 µL extracted DNA, with the following cycling program: 2 min at 94°C, 35 cycles of 30 s at 94°C, 30 s at 55°C and 30 s at 72°C, and a final extension step at 72°C for 5 min.

### Real-time qPCR to detect PCV2 nucleic acid in different organs/tissues

The experimental piglets were sacrificed at the end of the trial at 35 DPC, and various organs/tissues were collected for detection of PCV2 nucleic acid. Viral DNA was extracted from the samples using a DNA extraction kit (Tiangen) according to the manufacturer's instructions. For quantification of the PCV2 genome, a real-time qPCR technique established in this laboratory was performed (data not published). The sequence of primers was the same as mentioned above, and the TaqMan probe was 5′-FAM-ATGTATGTACAATTCAGAGAATTTA-TAMRA-3′ (1087–1063 nt). The thermal profile for the SYBR Green PCR was 95°C for 10 min, followed by 40 cycles of 95°C for 5 s, 61°C for 15 s and 72°C for 20 s. The results of the qPCR were expressed as the logarithm of the copies of PCV2 genome per gram sample (Lg copies/gram).

### Pathological studies

The macropathological lesions (i.e. the pathological condition) and the micropathological lesions (i.e. histological lesions) were considered together in the pathological studies. During postmortem examination, the pathological condition was observed, including ascites or edema, icterus, pale or congested liver, atrophy of lymph nodes, and enlargement or hemorrhage of peripheral lymph nodes. Each pathological condition was scored from 0 (none) to 2 (severe). At postmortem examination, samples were obtained from inguinal, submandibular and mesenteric lymph nodes, tonsil, spleen and lung and fixed by immersion in 10% buffered formalin. The fixed samples were dehydrated and embedded in paraffin wax in a single block per animal. Sections 4 µm thick were cut from each block. Each section was processed for routine histopathology and stained with hematoxylin and eosin. The pathological studies had two objectives. The first was to establish the diagnosis of PMWS at the moment of occurrence of clinical signs compatible with the disease, as reported previously [34]. In such a scenario, a pig was diagnosed with PMWS when all three criteria of the accepted international individual case definition for the disease (presence of clinical signs, mainly wasting, plus moderate to severe lymphoid lesions) were present [35]. Second, different pathological scores were established to assess potential differences in lymphoid tissue and microscopic lung lesions among the groups. Each type of histological lesion (lymphocyte depletion, granulomatous inflammation and alveolar walls with thickening and inflammatory infiltration) was scored from 0 (no lesions detected) to 3 (severe lesions detected) [36]. Therefore, the scores for the histological lesions were considered as follows: lymphocyte depletion [from 0 (none) to 3 (severe)], granulomatous inflammation [from 0 (none) to 3 (severe)], and alveolar wall inflammatory infiltration [from 0 (none) to 3 (severe)]. The clinical examinations of pathological condition and histological lesions were performed at 35 DPC. These parameters were evaluated in the experimental piglets by a blinded researcher and are summarized in Table 1.

### FCM measurements of leukocyte subpopulations in peripheral blood

Peripheral blood leukocyte subpopulations were measured by FCM. Murine monoclonal antibodies, which reacted with the porcine leukocyte CD antigens, were used. The antibodies included R-phycoerythrin (R-PE)-conjugated anti-SWC3a (4525-09; Southern Biotech, Birmingham, AL, USA), fluorescein-isothiocyanate-conjugated anti-CD4a (4515-02; Southern Biotech), and SpectralRed-conjugated anti-CD3ε (4510-13; Southern Biotech) and R-PE-conjugated anti-CD8a (4520-09; Southern Biotech). Monocytes and granulocytes in peripheral blood were measured by single-color FCM analysis using anti-SWC3a-R-PE, as described previously [10], [37]. For each sample, 10,000 cells were analyzed using an EPICS Elite flow cytometer and Expo32 software (Beckman–Coulter, USA). The proportions of monocytes and granulocytes in peripheral blood were calculated at different time points post-challenge to evaluate changes in the leukocyte subpopulations. For more detailed differentiation of T-cell subpopulations, blood mononuclear cells were analyzed using CD3/CD4/CD8 triple-color FCM analysis, which was performed as described previously [38], [39] with minor modification. For each sample, 10,000 cells were analyzed. Among the T-cell subpopulations, the proportion of T-helper cells (CD3^+^CD4^+^CD8^−^, CD4^+^) and cytotoxic T cells (CD3^+^CD4^−^CD8^+^, CD8^+^) was converted to the CD4^+^/CD8^+^ ratio to reveal changes in the immune response.

### Sequencing of the viruses from samples from the challenge groups

To confirm that the PCV2 virus was the same as that used for challenge in the different groups, respectively, the three different full-length PCV2 PCR products were amplified and sequenced from the DNA extracted from the inguinal lymph nodes, using a method described previously [17], [20].

### Statistical analysis

Comparisons of single treatment among different challenge groups (ADWG, qPCR and FCM results) were all performed using a nonparametric one-way ANOVA followed by LSD multiple comparison. The statistical analysis of the data was performed using SAS for Windows version 9.2 (SAS Institute, Cary, NC, USA). P<0.05 was considered statistically significant in all cases.
